# Sindbis virus self-amplifying replicon is compatible with modified nucleotides mediating expression and vaccine responses *in vivo*

**DOI:** 10.1016/j.omta.2026.201789

**Published:** 2026-06-18

**Authors:** Hiva Azizi, Gerard Agbayani, Tyler M. Renner, Bryan Simard, Umar Iqbal, Renu Dudani, Yimei Jia, Michael J. McCluskie, Bassel Akache

**Affiliations:** 1 National Research Council Canada, Human Health Therapeutics, Ottawa, ON K1A 0R6, Canada

**Keywords:** self-amplifying RNA, self-replicating RNA, modified nucleotides, alphavirus, VEEV, Sindbis, SINV, RNA vaccines, RNA therapeutics, lipid nanoparticles

## Abstract

The advent of effective formulations based on RNA encapsulated within lipid nanoparticles has led to the evaluation of this technology for its ability to treat and prevent a wide range of diseases. Expansion of the RNA-based tool kit available to vaccine and drug developers will accelerate this development and hopefully produce more robust and cost-effective therapies. With its more sustained expression profile, self-amplifying RNA offers many advantages to standard messenger RNA, allowing for the administration of lower RNA doses *in vivo* and potentially reducing dosing frequency for indications where long-term expression of a protein is required. We generated self-amplifying RNA containing the viral replicon from Sindbis virus, which demonstrated robust protein expression *in vitro* and *in vivo*. In addition, it showed increased compatibility with a number of modified nucleotides when compared to an equivalent RNA containing the replicon from Venezuelan equine encephalitis virus. These Sindbis-based self-amplifying RNAs containing modified nucleotides have the potential to be used in a variety of experimental and therapeutic applications.

## Graphical abstract

**Figure:**
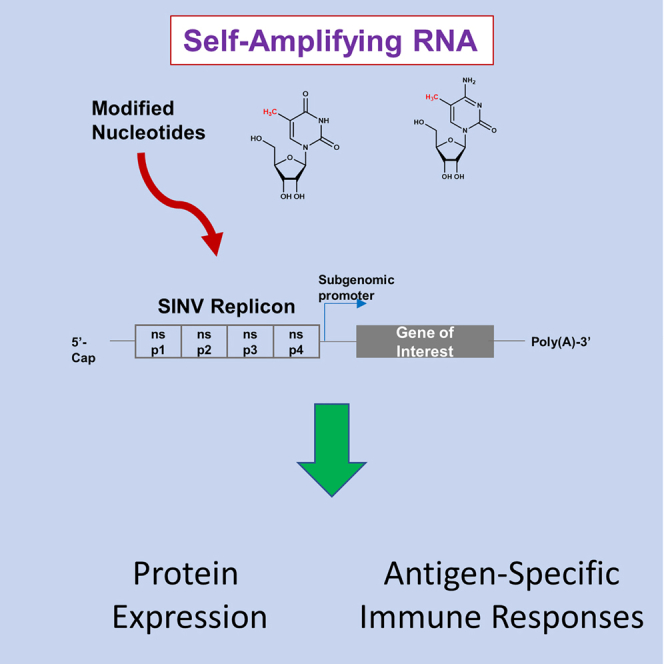

## Teaser

Lipid nanoparticle delivery of self-amplifying RNA (saRNA) instead of standard mRNA enables the use of lower doses and results in longer-lasting protein expression. A Sindbis-virus-based replicon is compatible with multiple modified nucleotides, resulting in saRNAs that mediate strong protein expression and immunogenicity, supporting further evaluation of their therapeutic potential.

## Introduction

Self-amplifying mRNA (saRNA) is a class of RNA-based vaccines/therapeutics that can generate prolonged expression profiles *in vivo* through continued RNA production post-delivery by employing viral replicon machinery derived generally from an alphavirus, such as Semliki Forest virus (SFV), Sindbis virus (SINV), or most commonly Venezuelan equine encephalitis virus (VEEV).^1^ Through RNA-dependent RNA polymerase (RdRp) function, the replicase drives robust production of full-length genomic RNA as well as subgenomic copies encoding the gene of interest, resulting in sustained high expression of encoded protein.^2^ Due to its self-replicating nature, saRNA vaccines can elicit equal or stronger adaptive immune responses to mRNA vaccines, at substantially lower dose, thus potentially lowering cost and turnaround time of manufacturing.^3^^,^^4^ In fact, the recent approval of the first VEEV-based saRNA COVID-19 vaccine Kostaive (ARCT-154) in Japan and EU reiterates the potential of saRNA as a potent vaccine platform.^5^ Indeed, the data from clinical trials showed that ARCT-154 elicited sustained, durable immune responses comparable to conventional mRNA.^5^ In addition, for therapeutic applications, the prolonged expression window of saRNA as compared to standard mRNA (weeks vs. days)^3^^,^^6^ could reduce the frequency of administration required to sustain a therapeutic benefit.

Despite the advantages it offers, the current saRNA platform can be further improved for clinical use, especially with regard to its tolerability, as a maximum tolerated dose reported for ARCT-121 (first iteration of ARCT-154) was only 7.5 micrograms.^7^ While the overall tolerability profile would be influenced by both the nature of the RNA and lipid formulations used to encapsulate them, the use of modified nucleotides, namely N1-methylpseudouridine (m1Ψ), has unlocked the full potential of standard mRNA vaccines, increasing their stability and importantly reducing their inflammatory profile.^8^^,^^9^^,^^10^ Similarly, modifying the chemistry of synthetic saRNA molecules could lead to improved activity/stability *in vivo*, especially as the size of saRNA based on alphaviruses reaches at least 8 kb, with the viral replicase open reading frame (ORF) alone exceeding 7 kb. This increased molecular length renders the saRNA backbone highly vulnerable to degradation, whether by intrinsic hydrolysis, physical breakdown, or chemical degradation from the lipid components when encapsulated within lipid nanoparticles (LNPs).^11^^,^^12^ To address these challenges, we and others have previously demonstrated the compatibility of certain modified nucleotides with the VEEV-based replicon, showing that complete substitution of the canonical equivalent with 5-hydroxymethylcytidine (5hmC), 5-methylcytidine (5mC), or 5-methyluridine (5mU) could result in stronger and/or more durable expression.^13^^,^^14^^,^^15^ These examples clearly illustrate that the use of modified nucleotides in the delivered synthetic saRNA is sufficient to change its activity profile *in vivo*, despite the fact that all subsequent genomic and subgenomic RNA molecules generated by the alphaviral replicase would presumably be composed of canonical nucleotides. Interestingly, VEEV-based saRNA has been shown to be incompatible with majority of modified nucleotides tested, including m1Ψ, the nucleotide used in clinical mRNA vaccines.^16^^,^^17^ This is likely due to the inability of the replicase enzyme to utilize this modified RNA as a template for RNA amplification. It is unknown if replicases from other alphaviruses would have similar profiles, with the exception of an SFV replicon generated with m1Ψ, which was reported to retain relative functionality, though unable to induce an immune response when tested in a mouse model.^18^ Herein, we report for the first time to our knowledge, the use of modified nucleotides with a replicon derived from SINV, showing that it is compatible with a larger number of modified nucleotides than its VEEV-based equivalent. These SINV saRNA with modified nucleotides induced protein expression as well as antigen-specific immune responses in *in vivo* models.

## Results

### SINV replicon drives robust expression of GFP reporter RNA containing canonical or modified nucleotides

**Figure 1:**
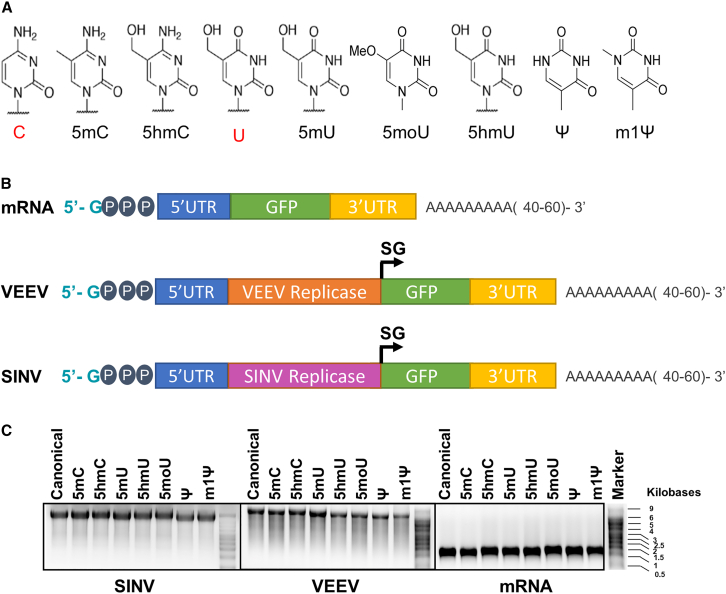
Schematic of the modified nucleosides and mRNA/saRNA platforms used in this study (A) Chemical structure of the seven modified nucleosides chosen for evaluation along with their canonical cytidine and uridine equivalents. (B) Schematic representation of the structure of GFP-encoding mRNA, VEEV, and SINV saRNA. (C) Formaldehyde agarose gel electrophoresis of GFP mRNA/saRNA samples produced with various nucleotide chemistries.

**Figure 2:**
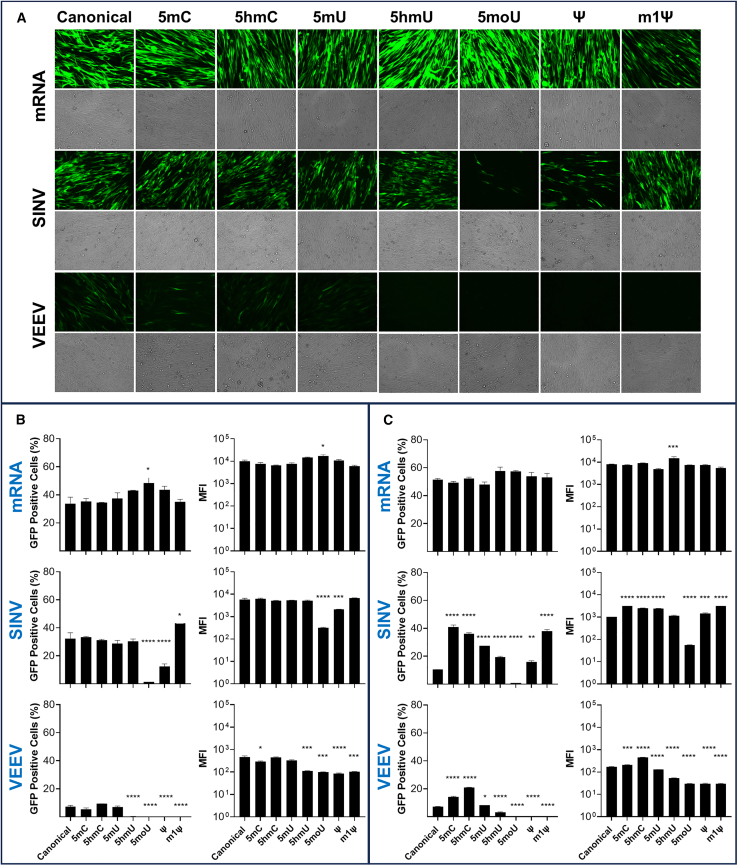
*In vitro* expression profile of mRNA/saRNA GFP samples generated with modified nucleotides (A) Fluorescent microscopy of BHK21 cells, 24 h post-transfection with GFP mRNA, SINV saRNA, and VEEV saRNA. Representative images of each condition when taken under brightfield (bottom) or with a GFP filter (top) are shown. (B) Subsequently, BHK21 cells were detached with PBS + 1% bovine serum albumin and 5 mM EDTA, fixed, and analyzed on an LSR II Fortessa. After gating on cells and singlets, the percentage of GFP-positive cells and their geometric mean fluorescence intensity was determined (*n* = 3 per condition). (C) Similar flow cytometrical analysis was conducted on HEK293T cells similarly transfected with RNA (*n* = 3 per condition). Grouped data for (B and C) are presented as mean +standard error of the mean (SEM). Results shown are representative of multiple experiments. Statistical significance of differences when compared to the canonical RNA are shown: ∗*p* < 0.05, ∗∗*p* < 0.01, ∗∗∗*p* < 0.001, and ∗∗∗∗*p* < 0.0001 by one-way ANOVA followed by Dunnett’s multiple comparisons test.

Seven modified nucleotide derivatives of cytidine or uridine, namely 5mC, 5hmC, 5mU, 5hmU, 5moU, pseudouridine (Ψ), and m1Ψ (Figure 1A), were selected for compatibility testing with SINV-based saRNA similar to previous studies with mRNA or VEEV saRNA.^9^^,^^10^^,^^14^^,^^17^^,^^18^ We sought to directly compare the impact of these modified nucleotides on protein expression using a GFP reporter in three different RNA formats: (1) mRNA, (2) VEEV saRNA, or (3) SINV saRNA (Figure 1B). Following *in vitro* transcription of each backbone with either all canonical nucleotides or with a selective substitution of a nucleotide with a respective modified counterpart, the integrity of the transcripts was confirmed through denaturing agarose gel electrophoresis (Figure 1C). Subsequently, 1 μg of RNA was transfected into BHK21 or HEK293T cells, seeded at 2 × 10^5^ cells per well, 16 h prior to transfection. Transfection efficiency was visually confirmed by fluorescent microscopy (Figure 2A; Figure S1). Cells were then detached and analyzed by flow cytometry to determine the percentage of GFP-expressing cells as well as their mean fluorescence intensity (MFI) (Figures 2B and 2C). An example of the flow cytometry dot plots is also shown (Figure S2). As expected, at one day following transfection, standard mRNA was capable of mediating strong protein expression when containing any of the modified nucleotides with >30% GFP+ cells measured for all mRNAs tested in both cell lines. As for VEEV-based saRNA, we were able to confirm previous reports that it was more sensitive to modified RNA chemistry.^13^^,^^14^ Complete substitution of uridine with the non-canonical nucleotides 5moU, Ψ, or m1Ψ yielded poorly functional saRNA, with <1% of cells classified as GFP+. Inclusion of 5mC, 5hmC, and 5mU generated similar or better expression as the purely canonical saRNA. Meanwhile, 5hmU-containing VEEV saRNA had detectable levels of GFP in HEK293T cells but only background levels of expression in the BHK21 cell line (Figures 2A, 2B, and 2C). Interestingly, saRNA containing the SINV replicon induced protein expression with a wider range of modified nucleotides, with 5mC, 5hmC, 5mU, 5hmU, or m1Ψ yielding similar or better levels of expression than the all-canonical control in both cell lines. Inclusion of 5moU, and to a lesser extent Ψ, had a negative impact on protein expression levels but was shown to be above background in at least one of the tested cell lines.

### Expression of modified nucleotide-SINV replicon encoding luciferase *in vivo*

**Figure 3:**
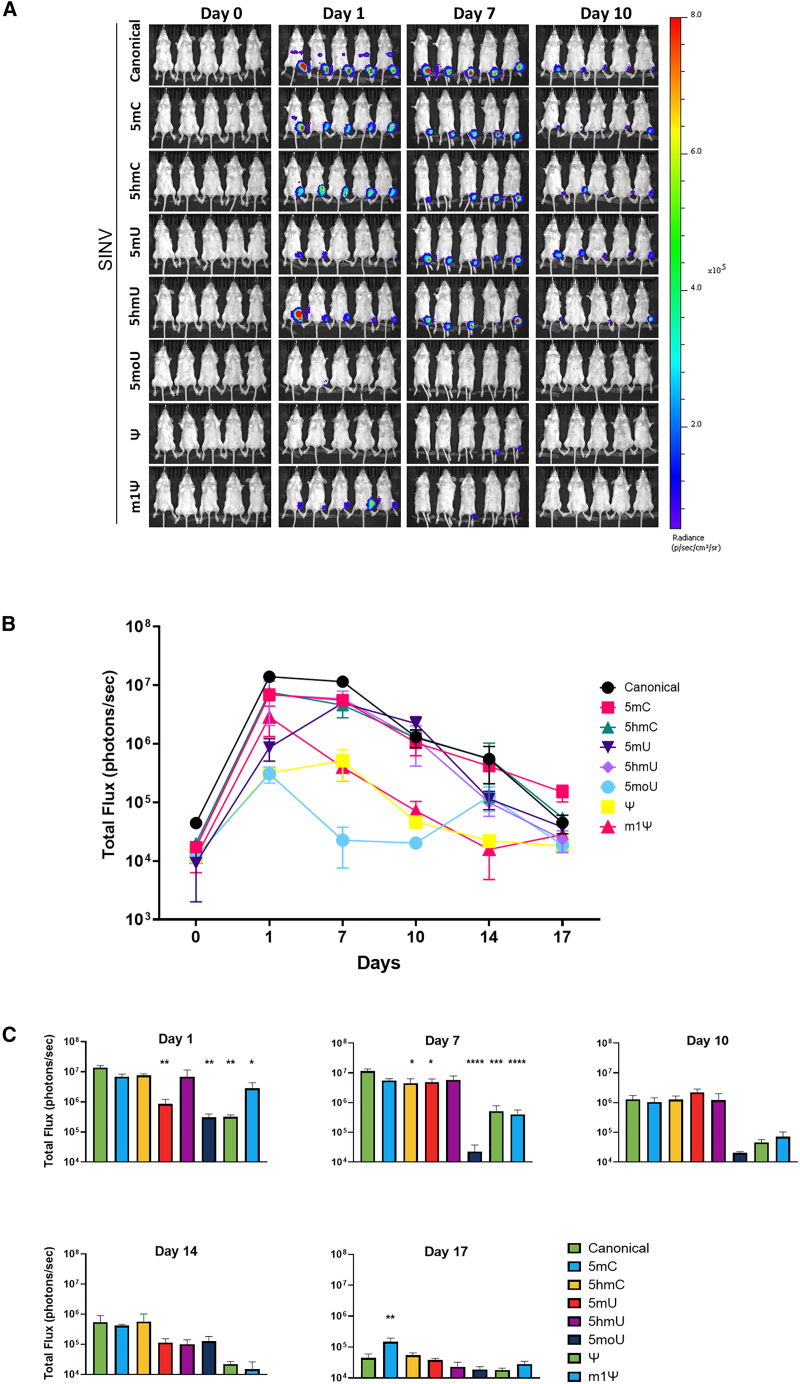
SINV saRNA generated with modified nucleotides exhibit robust expression *in vivo* C57BL/6 Albino mice (*n* = 5 per group) were injected i.m. with 2 μg canonical, 5mC-, 5hmC-, 5mU-, 5hmU-, 5moU-, Ψ-, or m1Ψ-modified SINV luciferase saRNA encapsulated within LNPs. (A) Whole body imaging was conducted to measure luciferase expression at various time points. (B) Total luminescence signals obtained from an ROI of the injection site were quantified and graphed to show total flux over time (group mean ± SEM). (C) The same data from Figure 2B are shown in bar graphs, separated by time point (group mean + SEM). Statistical significance of differences when compared to the canonical saRNA is shown: ∗*p* < 0.05, ∗∗*p* < 0.01, ∗∗∗*p* < 0.001, and ∗∗∗∗*p* < 0.0001 by one-way ANOVA followed by Tukey’s multiple comparisons test.

To determine if the compatibility of SINV replicon with modified nucleotides seen *in vitro* translated to the *in vivo* system, we generated LNP encapsulating SINV-based saRNA encoding the reporter gene luciferase. Albino C57BL/6 mice were injected with 2 μg of RNA intramuscularly. SINV saRNA containing either 5mU, 5mC, 5hmC, or 5hmU showed largely similar expression levels and kinetics as the canonical SINV saRNA, despite some statistically significant differences seen at some of the time points (Figures 3A, 3B, and 3C). As seen *in vitro*, inclusion of 5moU or Ψ significantly impacted the SINV saRNA’s ability to express luciferase, with levels similar to background seen throughout the study. Interestingly, the m1Ψ-containing luciferase SINV saRNA also appeared to induce weaker levels of expression, despite being able to generate similar levels of GFP protein *in vitro* (Figure 2). This was not due to differences in the genetic sequence, as comparable expression levels of luciferase protein were generated in HEK293T cells *in vitro*, with SINV saRNA containing 5mC, m1Ψ, or all canonical nucleotides (Figure S3).

### Immunogenicity of modified nucleotide-SINV replicon encoding model antigen ovalbumin *in vivo*

Finally, we sought to evaluate the ability of the SINV saRNA to induce antigen-specific humoral and cellular immune responses in mice. The model antigen ovalbumin (OVA) was selected due to its immunogenic profile and also to allow for direct comparison with a previous study using modified nucleotide VEEV saRNA.^13^ LNP formulations encapsulating OVA SINV saRNA with either canonical or modified nucleotide chemistry were generated. As controls, LNPs with OVA-encoding m1Ψ mRNA or canonical VEEV saRNA were also made. C57BL/6 mice (*n* = 5 per group) were injected with 2 μg of these RNA/LNP formulations intramuscularly on days 0 and 21. To assess the general tolerability of these formulations, the body weights and temperatures were measured for up to 1 week following the first or second vaccine dose. Mice in all groups appeared to gain weight similarly over the course of the study, with no significant differences in body weight seen vs. the naive control, except at day 22, when the mice receiving the Ψ saRNA formulation showed a statistically significantly lower gain in body weight (*p* < 0.05; Figure S4). As for body temperature, some minor statistically significant fluctuations were observed vs. the baseline measurements at day 0: canonical SINV saRNA at day 26 (2.3%; *p* < 0.05), m1Ψ mRNA at day 1 (−1.5%; *p* < 0.05) and day 22 (2.2%; *p* < 0.01), and naive animals at day 22 (2.2%; *p* < 0.05). As these differences were relatively minor, isolated, and also seen in the naive control group, it is unclear whether these were due to the formulations administered or due to statistical variance.

**Figure 4:**
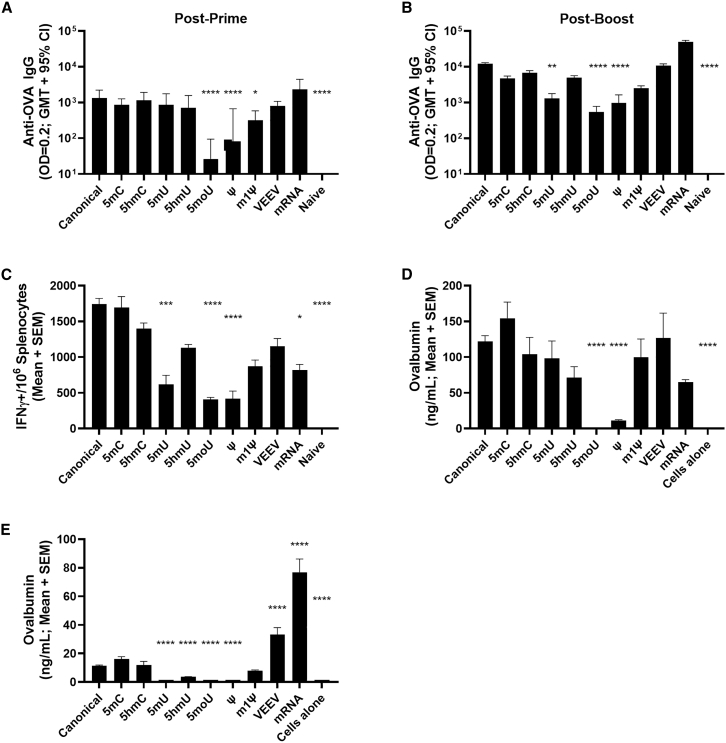
SINV saRNA generated with modified nucleotides induce antigen-specific immune responses in mice LNPs encapsulating canonical, 5mC-, 5hmC-, 5mU-, 5hmU-, 5moU-, Ψ-, or m1Ψ-modified SINV OVA saRNA were prepared. As controls, similar LNP formulations were generated with OVA-encoding canonical VEEV saRNA or m1Ψ mRNA. C57BL/6 mice (*n* = 5 per group) were injected i.m. with 2 μg on days 0 and 21. Serum samples collected on days 20 (A) and 35 (B) were analyzed by ELISA against ovalbumin protein to determine the antigen-specific IgG antibody titers. Grouped data are presented as geometric mean + 95% confidence interval (CI). (C) Splenocytes harvested on day 35 were analyzed by IFN-γ ELISpot when stimulated by SIINFEKL peptide. Values obtained with media alone were subtracted from those measured in the presence of the peptide. Grouped data are presented as mean + standard error of mean (SEM). OVA protein levels in HEK293T (D) or A549 (E) cell supernatants following overnight transfection by LNPs. The statistical significance of differences when compared to the canonical SINV saRNA: ∗*p* < 0.05, ∗∗*p* < 0.01, ∗∗∗*p* < 0.001, and ∗∗∗∗*p* < 0.0001 by one-way ANOVA followed by Dunnett’s multiple comparisons test.

As for antigen-specific immune responses, OVA-specific immunoglobulin G (IgG) titers were measured in the sera following a single or double vaccine dose at days 20 and 35, respectively (Figures 4A and 4B). All vaccine formulations were immunogenic, with 5mC, 5hmC, and 5hmU SINV saRNA inducing similar titers as the canonical control. At both time points, 5moU and Ψ SINV saRNA induced weaker immune responses, with >12-fold lower anti-OVA IgG titers measured vs. the canonical SINV saRNA (*p* < 0.0001). The m1Ψ SINV saRNA induced a less-pronounced ∼4-fold decrease in titers vs. the canonical control at both measured timepoints, but this only reached a level of statistical significance at day 20 (*p* < 0.05). Interestingly, the antigen-specific titers in mice immunized with 5mU SINV saRNA were largely unchanged following boost (geomean titers [GMT] of 858 vs. 974 at days 20 and 35, respectively). This led to significantly lower titers at day 35 vs. the canonical control (*p* < 0.01), which had GMT of 1,327 and 11,924 at days 20 and 35, respectively. All vaccine formulations were able to induce antigen-specific CD8^+^ T cells recognizing the OVA immunodominant epitope SIINFEKL as determined by ELISpot on splenocytes collected at day 35 (Figure 4C). The number of spot-forming cells (SFCs) was highest in animals receiving the canonical or 5mC SINV formulations, with 1,741 and 1,692 interferon-gamma-positive (IFN-γ^+^) SFCs/10^6^ splenocytes, respectively. Again, responses were significantly lower with 5moU and Ψ SINV saRNA when compared to the canonical control (*p* < 0.0001). In addition, the 5mU formulation also showed a lower ability to mediate antigen-specific T cell responses (*p* < 0.001). As for the other RNA types, immunization with m1Ψ mRNA yielded significantly lower number of IFN-γ^+^ SFCs/10^6^ splenocytes vs. the canonical SINV saRNA (820 vs. 1741; *p* < 0.05), while VEEV and SINV saRNA induced statistically similar responses.

Finally, the LNPs were tested *in vitro* for their ability to mediate protein expression in HEK293T cells, as well as IFN-sensitive A549 cells. Following overnight transfection with the LNPs, cellular supernatant was collected to measure levels of OVA protein by sandwich ELISA. In HEK293T cells, most of the chemically modified SINV saRNA formulations were able to produce similar levels of OVA protein as the canonical nucleotide SINV saRNA (Figure 4D). Significantly lower levels of protein were measured with the Ψ and 5moU SINV saRNA (*p* < 0.0001), with the latter’s signal being below the assay’s lower limit of quantification. In addition, no significant difference in protein expression was seen in cells transfected with the canonical SINV saRNA, VEEV saRNA, or m1Ψ mRNA. Interestingly, in A549 cells, significantly lower protein production was observed with 5mU, 5hmU, as well as 5moU and Ψ SINV saRNA when compared to the canonical control (Figure 4E; *p* < 0.0001). To determine if the modified nucleotides would impact the kinetics of expression *in vitro*, supernatants were collected and analyzed from similarly transfected cells at 6 or 48 h post incubation with the LNPs (Figure S5). At 48 h, the trends in protein expression induced by the different RNA/LNP formulations in both HEK293T and A549 cells were largely similar to what was observed at the 24-h time point. At 6 h, no ovalbumin expression was detected with any of the SINV or VEEV saRNA/LNPs in the A549 cells, while in HEK293T cells, higher levels of protein expression were detected with the canonical SINV saRNA vs. any of the chemically modified saRNA (*p* < 0.0001). Northern blot analysis on RNA extracted from similarly transfected HEK293T cells revealed that the lack of detectable protein expression in cells transfected with 5moU SINV saRNA coincided with an absence of subgenomic RNA production (Figure S6). In samples showing detectable subgenomic RNA signals in the northern blot, it was also possible upon overexposure of the blot to detect larger RNA molecules that could correspond to the genomic RNA (data not shown). This indicates that both types of RNA are generated by the replicase *in vitro* but that production of the subgenomic RNA is strongly favored.

## Discussion

With their enhanced utility and versatility, RNA/LNP-based formulations hold great promise in advancing a new range of vaccines and therapies. Herein, we sought to expand the RNA toolbox from standard mRNA and VEEV-based saRNA by generating an SINV-replicon-based saRNA and evaluating its compatibility with a number of modified nucleotides. Our results demonstrate that saRNA containing the SINV replicase can indeed induce strong protein expression and antigen-specific immune responses. It also shows wider compatibility with the tested modified nucleotides than its VEEV-based counterpart, which could have important implications with regard to its stability, tolerability, and/or immunogenicity profiles.

As highlighted with the recent development of novel multivalent mRNA vaccines against seasonal influenza, it becomes more challenging to simultaneously administer sufficient doses of the multiple RNA types necessary to generate effective broad immunogenicity in the clinic, while limiting adverse events.^19^ Alternate synthetic RNA platforms, such as saRNA, have demonstrated advantages, such as conferring protection and inducing long-lasting immunity with a vaccine dose of one order of magnitude lower than that used with mRNA, while showing improved comparative tolerability.^5^ Despite the promising results from clinical and pre-clinical studies with saRNA vaccines, there remains room to improve their stability, activity, and/or safety profile, potentially expanding their use for different vaccines or therapeutic applications. In fact, the large size of the replicon can influence their susceptibility to degradation and storage. In addition to increasing stability of RNA molecules, the inclusion of modified nucleotides in VEEV-based saRNA has been shown to alter their activity profiles, enhancing the expression and/or immunogenicity of their cargo protein, while reducing reactogenicity of the vaccine.^13^^,^^14^^,^^15^^,^^17^ For example, VEEV-based saRNA with 5mC showed similar or superior immunogenicity to those containing canonical nucleotides, while inclusion of 5mU resulted in reduced immunogenicity but a longer expression window, ideal for therapeutic applications.^13^ However, the VEEV replicon was only shown to be compatible with a few modified nucleotides and not with m1Ψ.^13^^,^^14^^,^^15^^,^^17^ Studies conducted with the SFV replicase have shown that while a functional replicon can be generated with m1Ψ, it showed significantly impaired amplification capacity *in vitro* and *in vivo*.^18^

SINV-based replicons have not yet been tested clinically and have been used rarely in preclinical studies.^20^ A recent study compared the expression profile *in vivo* of SINV-based saRNA to a panel of other replicons based on other Old World as well as New World alphaviruses such as VEEV.^6^ They showed that the delivery platform can influence the duration and decay rate of protein expression mediated by the saRNA, with lower expression seen with SINV- vs. VEEV-based replicons when delivered by LNPs, but not when encapsulated with poly(cystamine bisacrylamide-co-4-amino-1-butanol) (pABOL) polymer. While differences in the level of GFP expression were seen between the VEEV and SINV saRNA in our studies *in vitro*, this could be due to a number of factors including the transfection methods used. This is supported by the fact that VEEV and SINV saRNA induced similar levels of OVA protein expression when delivered by LNP in an *in vitro* setting (Figure 4D). However, other variables such as the specific viral strains from which the replicases are derived (see Materials and Methods section below) as well as our use of a CleanCap-AG instead of the naturally occurring AU at the 5′ end of the saRNA could have impacted the expression profile of the VEEV saRNA. In future studies comparing the functionality and efficacy of VEEV vs. SINV saRNAs for a specific application, it will be important to use the optimized versions of each construct.

The incorporation of modified nucleotides into SINV-based saRNA molecules could increase their stability, activity, and tolerability profiles, enhancing their potential use for various clinical applications. Overall, our study highlights that SINV-based replicons are compatible with a wider range of modified nucleotides than VEEV, mediating protein expression *in vitro* with six of seven of the modifications we tested. In addition, certain modifications, namely 5mC, 5hmC, 5mU, and 5hmU demonstrated comparable expression profile *in vivo*. The Ψ- and m1Ψ-containing SINV-based replicon appears to induce lower expression in muscles *in vivo*. Interestingly, 5mC-, 5hmC-, and m1Ψ-containing SINV saRNA showed similar protein expression levels as the canonical version in both HEK293T and A549 cells *in vitro* (Figures 4D and 4E; Figure S5). Similar to results with m1Ψ-containing SFV-based saRNA,^18^ the incorporation of m1Ψ or these other modified nucleotides into SINV saRNA appeared to impact the early kinetics of protein expression, with lower expression measured vs. their canonical counterpart at 6 h post-transfection. The lower expression profile seen *in vivo* with m1Ψ-containing SINV saRNA could be due to the differences in their ability to induce innate immune signaling pathways *in vivo* and/or the cell types evaluated in our study *in vivo* vs. *in vitro* (muscle vs. kidney/lung, respectively). Protein expression profiles were severely impacted with 5mU and, to a lesser degree, 5hmU compared to canonical SINV saRNA in A549 cells. This indicates that they are more susceptible to inhibition of translation in an RNA-sensing competent environment. Although 5mU saRNA showed lower protein expression up to 7 days following delivery *in vivo* (Figure 3), this did not appear to have a large impact on protein expression at later time points. This could be due to the gradual replacement of the synthetic chemically modified RNA with newly replicated canonical RNA within the cells. As shown previously with VEEV vs. SINV saRNA *in vivo*, the delivery platform can impact magnitude and kinetics of expression.^6^ It would be interesting to investigate the expression profile in different cell types and tissues *in vivo* in future studies.

With regard to potential utility in a vaccine setting, the SINV saRNA with either canonical or modified chemistry were able to induce strong antigen-specific immune responses to the model antigen OVA. However, not all modified nucleotides behaved similarly. Incorporation of 5moU or Ψ in the SINV saRNA resulted in significantly lower antigen-specific IgG titers and T cells at all time points tested. The reduced immunogenicity of these formulations appeared to coincide with a reduced ability to generate OVA protein and subgenomic RNA *in vitro* (Figure 4D; Figure S6). SINV saRNA containing 5mU or m1Ψ showed weaker immunogenicity at certain time points or readouts, despite inducing similar levels of protein expression. In the case of 5mU, the pattern was very similar to what was observed with VEEV saRNA previously, where it was able to induce similar levels of both antigen-specific humoral and cellular responses as a canonical saRNA after a single dose, but immune responses were significantly lower following boost.^13^ Finally, the use of 5mC, 5hmC, or 5hmU SINV saRNA resulted in similar immunogenicity profiles as the canonical formulation. It would be interesting to evaluate these nucleotides with SINV saRNA encoding different antigens, as 5mC VEEV saRNA was shown to induce superior responses to SARS-CoV-2 spike.^14^ In our study, SINV saRNA induced a similar magnitude of antigen-specific immune responses as a canonical VEEV saRNA or m1Ψ RNA. Interestingly, despite producing similar levels of OVA protein *in vitro*, the SINV saRNA vectors generated higher levels of subgenomic RNA in the cells. It will be interesting to evaluate the impact of this higher RNA production on innate immune activation and other parameters in future studies. The SINV saRNA formulations containing the modified nucleotides that we tested *in vivo* appeared to be well tolerated with no observed adverse events or major changes in body weight/temperature (Figure S4), but a more in-depth safety/tolerability assessment will need to be conducted in the future with the specific vaccine or therapy being developed.

In conclusion, SINV-replicon-based saRNA containing modified nucleotides can be used to mediate protein expression *in vitro/in vivo*. This platform also demonstrates strong immunogenicity *in vivo*. This could have potential benefits for various applications, in either prophylactic vaccine settings as well as for different types of therapies. Future head-to-head studies will determine if SINV-saRNA-based therapies or vaccines offer any benefits with regard to efficacy, safety, and/or manufacturing when compared to the more established synthetic mRNA or VEEV saRNA platforms.

## Materials and methods

### Plasmid constructs and *in vitro* RNA transcription

VEEV saRNA plasmid templates used for *in vitro* transcription (IVT) of RNA has been described previously,^13^ with the replicase derived from the SCR725 plasmid (Sigma-Aldrich, St. Louis, MO, USA). Its sequence is identical to that from the TC-83 attenuated VEEV strain, except for the presence of a serine instead of a proline at amino acid 1308. Plasmid DNA template for messenger RNA (Invitrogen, Carlsbad, CA, USA) was modified by insertional mutagenesis to become compatible with co-transcriptional capping. Subsequently, mtRNR1-AES 3′ untranslated region (UTR) bearing poly-A tail was gene synthesized (IDT DNA, Coralville, IA, USA) and cloned via HindIII-XhoI sites, generating pcDNA-GFP plasmid (Table S1). For SINV, the replicon was derived from the infectious AR339 strain pTE3′2J amplified from pBG78 plasmid (Kerafast, Newmark, CA, USA). Then T7 promoter sequence compatible with CleanCap-AG was inserted next to the 5′- UTR using primers listed in Table S1 and an infusion cloning kit (Takara Bio, Saint Jose, CA, USA), and the 3′-end of nsp4 was then amplified using appropriate primers and pBG218 (Kerafast) as template. The resulting PCR product was then fused to GFP-3′ UTR, which had been PCR-amplified using primers 5 and 6 and pBG78 as template. The final product was then inserted into pBG78 plasmid backbone linearized with HpaI-ApaI, yielding SINV-GFP, optimized for IVT. To generate SINV-LUC and SINV-OVA replicons, 3′ end of nsp4 and ORFs were PCR-amplified using primers described in the table, then subjected to a fusion PCR reaction, followed by infusion cloning (Takara Bio) into SINV-GFP plasmid via HpaI-XbaI sites. The SINV-GFP plasmid used for IVT was linearized with PspOMI, while SINV-LUC and SINV-OVA were digested with PmeI prior to IVT. PcDNA-GFP plasmid was linearized by HindIII, while pcDNA-OVA and VEEV-OVA plasmids were linearized with XbaI. Preparation of DNA template for IVT reaction and subsequent purification steps were conducted as previously described.^13^ To assess RNA size and integrity, all RNA samples were resolved on formaldehyde (2.6% v/v) denaturing agarose (1% w/v) gel. Millenium RNA ladder (Thermo Fisher Scientific, Waltham, MA, USA) was used as the size marker.

### Lipid nanoparticle encapsulation of saRNA

To encapsulate RNA encoding luciferase or OVA into LNP, a commercially available kit based on SM102 was utilized (Cayman Chemical, Ann Arbor, MI, USA), and formulations were prepared using the NanoAssemblr Ignite (Precision NanoSystems, Vancouver, Canada) as per the manufacturer’s instructions.^13^ All formulations were filter-sterilized using 13 mm Acrodisc 0.2 μM syringe filters (Pall, NY, USA) and stored in 10% sucrose buffer and evaluated for quality control attributes, such as particle size and polydispersity index using the Zetasizer Nano ZS (Malvern Instruments, Malvern, UK).

### *In vitro* expression in BHK21, HEK293T, and A549 cells

For GFP expression analysis, cells (2 × 10^5^ per well) were transfected with 1 μg RNA using lipofectamine 3000 (Thermo Fisher Scientific) transfection reagent. Maintenance and transfection of HEK293T and BHK21 cells as well as fluorescent microscopy and flow cytometry analysis were carried out as previously reported.^13^ Briefly, cells were detached with PBS +1% bovine serum albumin and 5 mM EDTA, washed, fixed, and analyzed on the LSR II Fortessa (Becton Dickinson). Flow cytometry data were analyzed using FlowJo (Becton Dickinson). GFP transfection efficiency was determined based on the percentage of cells positive for GFP expression. Mean fluorescence intensity (MFI) + standard error of the mean (SEM) was used to measure overall GFP expression.

For measurement of OVA expression, the supernatant media was collected after incubation of HEK-293T (2.5 × 10^5^ cells per well) or A549 (1 × 10^5^ cells per well) cells with 1 μg of SM102 encapsulated RNA at various timepoints. Levels of OVA were measured using a commercial OVA ELISA kit (Abcam, Cambridge, UK) according to manufacturer’s instructions.

### Northern blot

To measure the replicative ability of the saRNA, HEK293T cells (3 × 10^6^ per T25 flask) were incubated with 5 μg of SM102 encapsulated OVA RNA overnight. Cellular RNA was isolated from the cells using TRIzol (Thermo Fisher Scientific) and resolved on a formaldehyde (2.6% v/v) denaturing agarose (1% w/v) gel prior to transfer to a Nytran SuperCharge membrane (Cytiva Life Sciences, Marlborough, MA, USA). RNA was cross-linked to membrane through exposure to UV light. To detect OVA-containing RNA, an RNA antisense probe covering the entire ORF was generated by IVT using a DNA template in which a T7 promoter was inserted on the negative strand at the 3′ end of the ORF. The IVT reaction was performed as above without the CleanCap -AG, with all canonical nucleotides, except for UTP where 35% of the nucleotides consisted of Biotin-16-AA-UTP (TriLink BioTechnologies, San Diego, CA) and 65% of canonical UTP. The probe was hybridized to the membrane for 4 h using the ULTRAhyb hybridization buffer and then detected by the chemiluminescent nucleic acid detection module (both from Thermo Fisher Scientific) according to manufacturer’s instructions. Imaging of the northern blot was conducted on ChemiDoc Imaging System (Bio-Rad, Hercules, CA, USA).

### Insitutional review board statement

Mice were maintained at the small animal facility of the National Research Council (NRC) Canada in accordance with the guidelines of the Canadian Council on Animal Care. All procedures performed on animals in this study were approved by our Institutional Review Board (NRC Human Health Therapeutics Animal Care Committee) and covered under animal use protocol 2024.14. All experiments were carried out in accordance with the ARRIVE guidelines.

### *In vivo* expression analysis

Female albino C57BL/6 mice (6–8 weeks old) were obtained from Charles River Laboratories (Saint-Constant, QC, Canada). Mice (*n* = 5 each group) were injected intramuscularly (i.m.) into left tibialis anterior muscle with 2 μg of LNP-encapsulated luciferase saRNA in a total volume of 50 μL. Fifteen minutes prior to whole body imaging, D-luciferin (PerkinElmer, Waltham, MA, USA) was injected subcutaneously at a dose of 0.15 mg per gram of mouse body weight. Imaging and data analysis were performed as previously described.^13^

### *In vivo* immunogenicity analysis

Female C57BL/6 mice (6–8 weeks old) were obtained from Charles River Laboratories (Saint-Constant, QC, Canada). Mice (*n* = 5 per group) were immunized by i.m. injection (50 μL) into the left tibialis anterior muscle on days 0 and 21 with 2 μg of encapsulated OVA RNA. Prior to and for 7 days following each immunization, mice were monitored for clinical signs (e.g., lethargy and piloerection), body temperature, and body weight. For body temperature, transponders (Model IPTT-300, BioMedic Data Systems Inc., Seaford, DE, USA) were implanted subcutaneously prior to study start using a 12-gauge needle, and the temperature was read in a non-invasive manner through the skin with a probe. On day 20, mice were anesthetized with isoflurane and bled via the submandibular vein for serum collection. On day 35, mice were bled as above for serum collection and then euthanized by cervical dislocation prior to collection of spleens for measurement of cellular immune responses by IFN-γ ELISpot, targeting the well-recognized CD^8+^ T cell epitope OVA_257-264_: SIINFEKL (JPT Peptide Technologies GmbH, Berlin, Germany), as previously described.^21^ Anti-OVA IgG levels were determined by ELISA as previously described.^22^ Samples were also collected from naive animals to establish background immune response levels. Each individual mouse sample was analyzed separately across the various readouts.

### Statistical analysis

Data were analyzed using GraphPad Prism v.10 (GraphPad Software, Boston, MA, USA). Statistical significance of the difference between groups was calculated by one-way or two-way ANOVA, as indicated in the figure legends. Data were log transformed for ovalbumin/IgG ELISA and IFN-γ ELISpot prior to statistical analysis. For all analyses, differences were considered to be not significant with *p* > 0.05. Significance was indicated in the graphs as follows: ∗*p* < 0.05, ∗∗*p* < 0.01, ∗∗∗*p* < 0.001, and ∗∗∗∗: *p* < 0.0001.

## Data and code availability

The original contributions presented in the study are included in the article/supplemental materials, and further inquiries can be directed to the corresponding author.

## Acknowledgments

The authors would like to acknowledge all of the staff of the Human Health Therapeutics Animal Resource Group for their excellent technical support of the animal studies. Project was funded internally by the National Research Council Canada.

## Author contributions

H.A. and B.A. took the lead in writing the manuscript. H.A., G.A., T.M.R., B.S., U.I., R.D., Y.J., and B.A. designed/carried out the experiments or analyzed the data. M.J.McC. and B.A. oversaw the project. All authors contributed to the article and approved the submitted version.

## Declaration of interests

The authors declare no competing interests.

## Supplemental information

Document S1. Figures S1–S6 and Table S1

Document S2. Article plus supplemental information
