# Supplementary material for: Sindbis virus self-amplifying replicon is compatible with modified nucleotides mediating expression and vaccine responses in vivo
Source: Mol Ther Adv. 2026 Jun 18;34(3):201789. doi: 10.1016/j.omta.2026.201789 (PMC13355167; doi:10.1016/j.omta.2026.201789)
Supplement: Document S1. Figures S1–S6 and Table S1 [file mmc1.pdf]

OMTA, Volume *34*

Supplemental information

Sindbis virus self-amplifying replicon is compatible with modified nucleotides mediating expression and vaccine responses *in vivo*

Hiva Azizi, Gerard Agbayani, Tyler M. Renner, Bryan Simard, Umar Iqbal, Renu Dudani, Yimei Jia, Michael J. McCluskie, and Bassel Akache

Table S1

List of primers used to create plasmid templates for in vitro transcription of RNA.

	Primer	Sequence	Product	Construct
1	T7AG-SINV-5UTR-Infusion-F	TAATACGACTCACTATAAGATTGACGGCGTAGTACACACT	T7AG-pBG78	T7AG-pBG78
2	T7AG-SINV-5UTR-Infusion-R-vF	TTATAGTGAGTCGTATTAGGTTCACTAAACGAGCTCTGCT		
3	nsp4-HpaI-Infusion-F	GACGACGCTATGGCGTTAACCGGTCTGATGATCTTGGA	Nsp4-3'end	pSINV-EGFP
4	nsp4-SG-R-fusion-R	ATGAAATGTACTATGCTGACTATTTAGGACCACCGTAGAGAT		
5	SG-fusion-EGFP-F	TAGTCAGCATAGTACATTTCATCTGACTAATACTACAACACCACCACCATGGTGAGCAAGGGCGAGGA	EGFP-3'UTR	
6	pBG78-PolyA-delHDVR-Infusion-R	ATCAGCGGGTTTAAACGGGCCCTTTTTTTTTTTTTTTTTTTTTTTTTTTTTTTTTTTTTTTTTTTTTTTTTTTTTTTTGAAATGTTAAAAACA		
7	SINV-HpaI-Infusion-F	AAGACGACGCTATGGCGTTAACCGGTCTGATGATCTTGGA	SINV nsp4-3'	pSINV-LUC
8	SINV-SG-fusion-R	GGTGGTGGTGTTGTAGTATTAGT		
9	SINV-SG-CBR2opt-fusion-F	ACTAATACTACAACACCACCACCATGGTTAAGAGAGAAAAAAATGT	LUC	
10	SINV-XbaI-CBR2opt-Infusion-R	AGCGTCTAGGATCCATGGTCTAGATCATTAAACGCCGCCAGCCTTAAC		
11	SINV-SG-OVA-Fusion-F	ACTAATACTACAACACCACCACCATGGGCTCCATCGGTGCA	OVA	pSINV-OVA
12	SINV-XbaI-OVA-Infusion-R-V2	AGCGTCTAGGATCCATGGTCTAGATTAAGGGGAAACACATCTGCCA		
13	pcDNA-T7AG-Insertion-F	TAATACGACTCACTATAAGGGAATAAACTAGTATTCTTCTGGT	pcDNA-T7AG	pcDNA-T7AG
14	pcDNA-T7AG-Inseryion-R	ACCAGAAGAATACTAGTTTATTCCC		
15	pcDNA-TagGFP2-Infusion-F	TGATCATAAGAAGACCAGGGGTGTCTGAGTCTCTCTTGGGCGGTGGTACCACTCGCCCCCGCTCCTCGA	TagGFP2	pcDNA-T7AG-TagGFP2
16	pcDNA-TagGFP2-Infusion-R	TGCATGCAGTACCAGCTCGAGTTACCTGTACAGCTCGTCCAT		

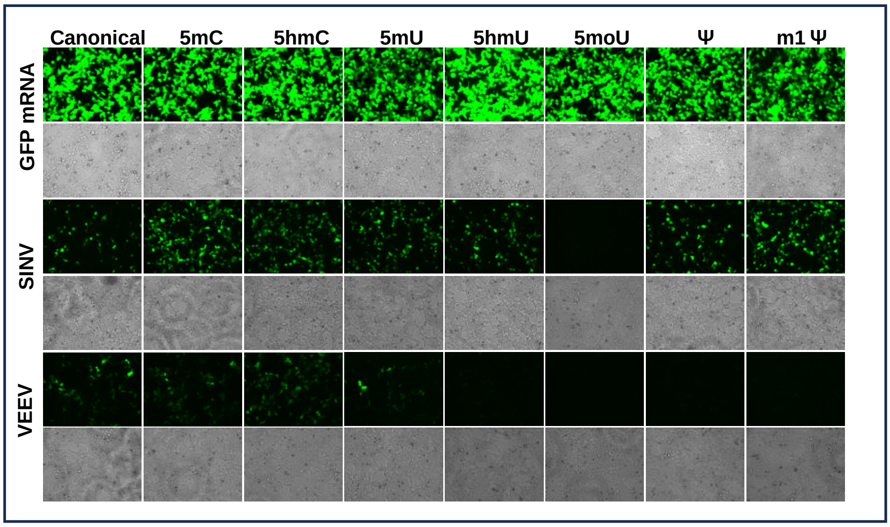

Figure S1

Fluorescent microscopy of HEK293T cells at 24 hours post transfection with GFP-encoding mRNA, SINV saRNA or VEEV saRNA. Cells were also analyzed by flow cytometry to determine percentage positivity for GFP expression as well as mean fluorescence intensity (see Figure 2C).

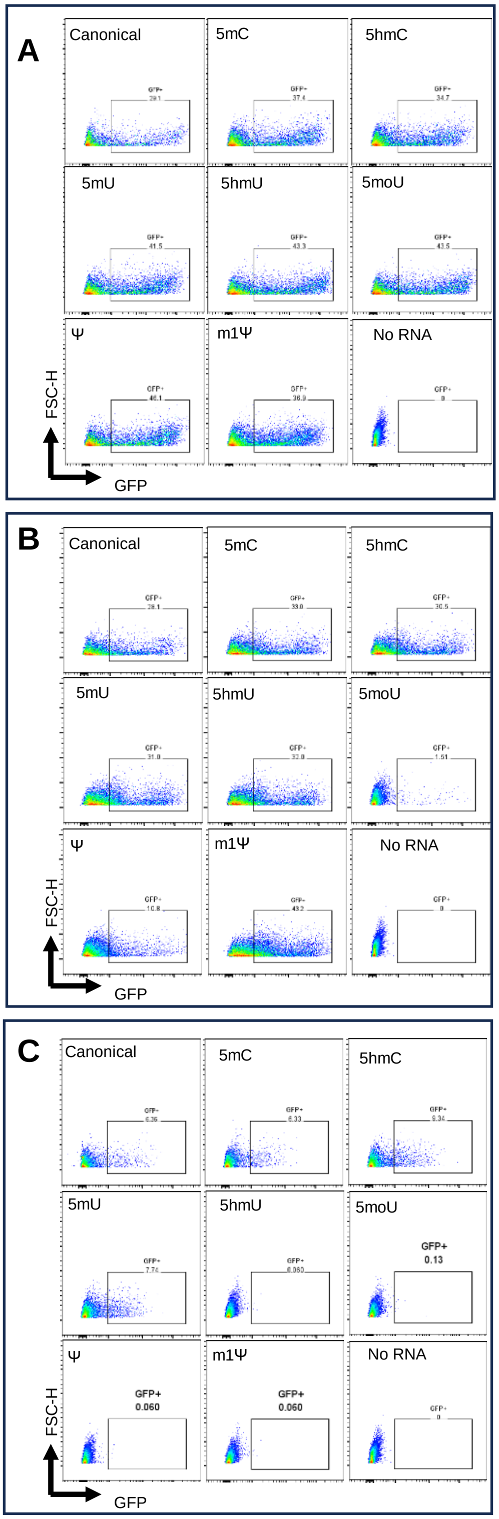

Figure S2
Flow cytometry dot blots of BHK cells transfected with canonical or chemically modified A) mRNA, B) SINV saRNA or C) VEEV saRNA.

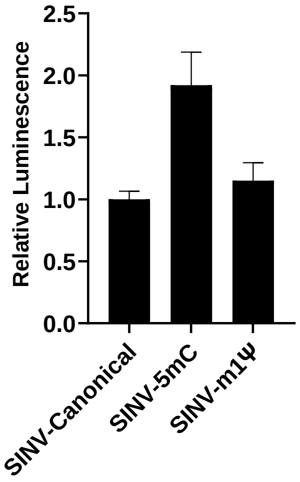

Figure S3
SINV luciferase saRNA expression *in vitro*. SINV saRNA generated with canonical, 5mC or m1Ψ nucleotides were transfected into HEK293T cells, and bioluminescent signal was measured at 24h post-transfection using Bright-Glo assay kit (Promega, USA), as per the manufacturer’s instructions.

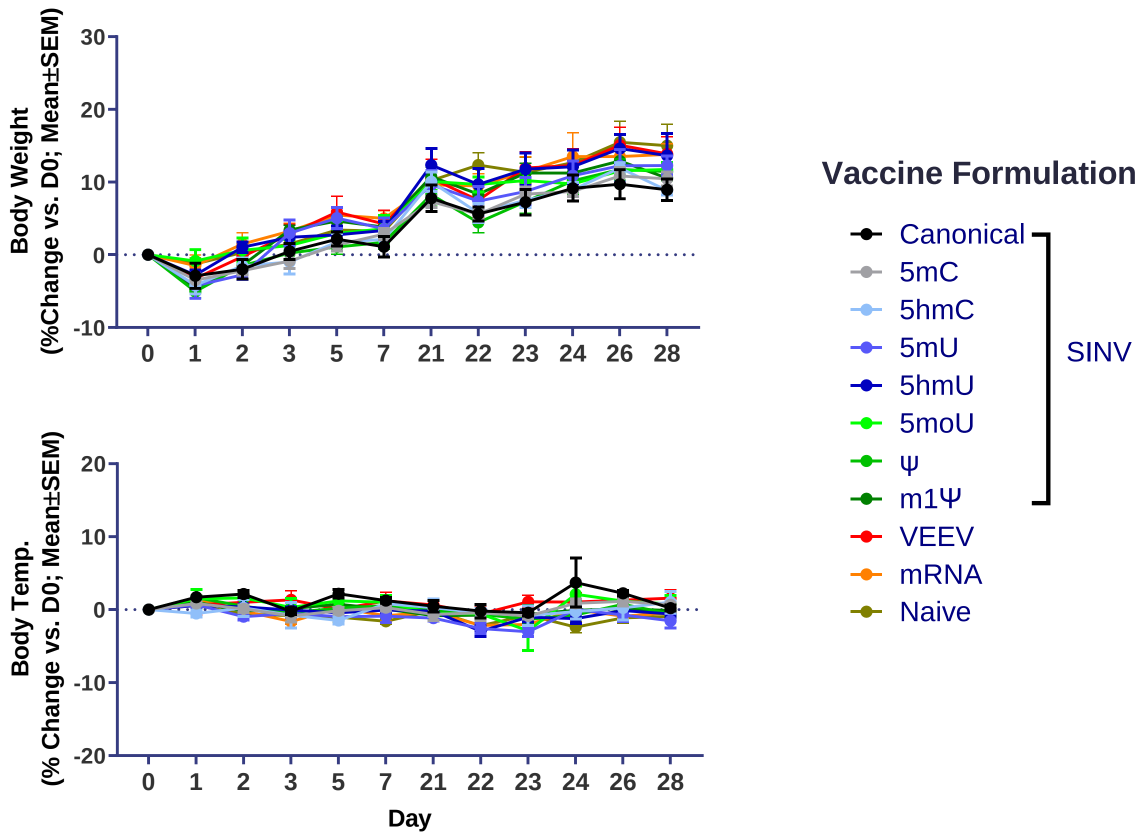

Figure S4

Tolerability of OVA/LNP vaccine formulations in mice. Vaccinated mice described in Figure 4 were monitored for changes in body weight (A) and temperature (B) up to 7 days following each vaccination. Grouped data are presented as mean ± standard error of mean (SEM).

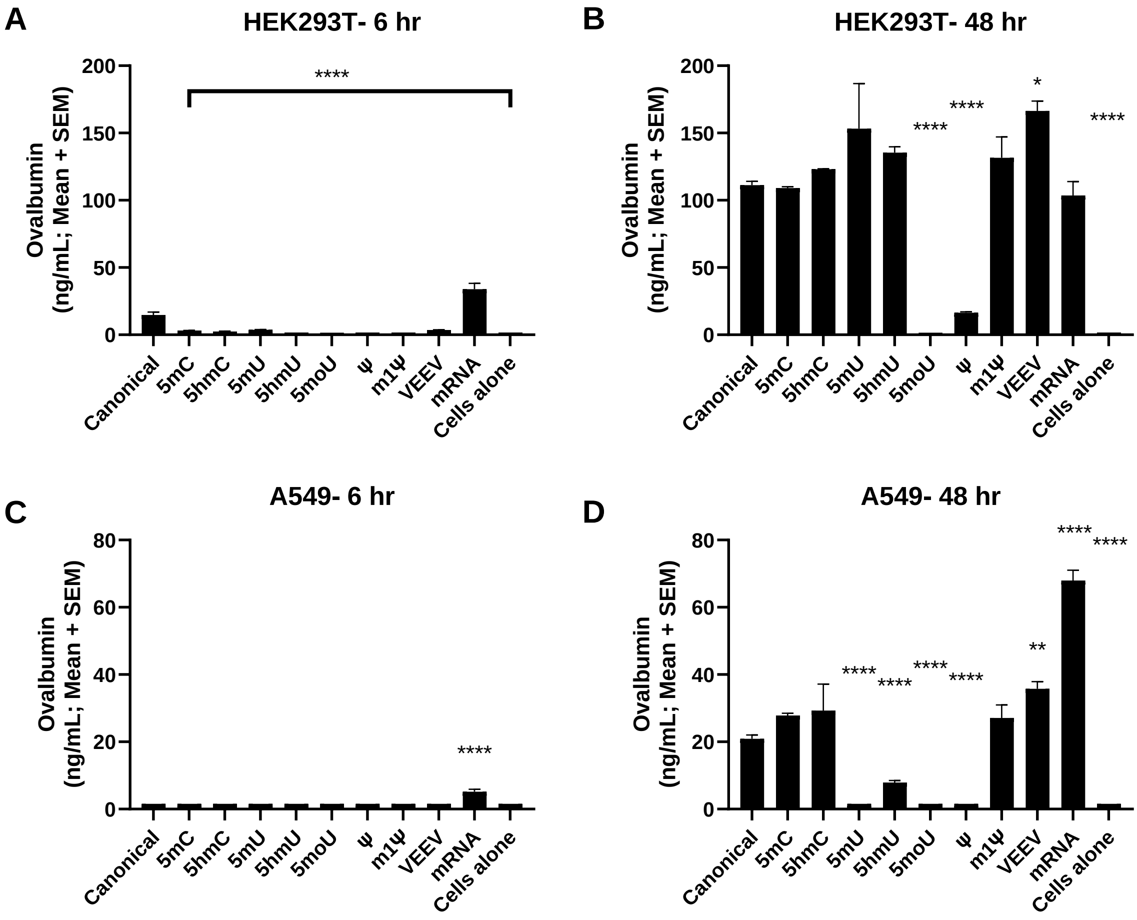

Figure S5

OVA protein expression in HEK293T and A549 cells in vitro. OVA RNA/LNP formulations described in Figure 4 were transfected into HEK293T and A549 cells, and cellular supernatant collected at 6 or 48 hours post transfection for measurement of OVA protein levels by sandwich ELISA. The statistical significance of differences when compared to the canonical SINV saRNA: *: $p < 0.05$ and ****: $p < 0.0001$ by one-way ANOVA followed by Dunnett's multiple comparisons test.

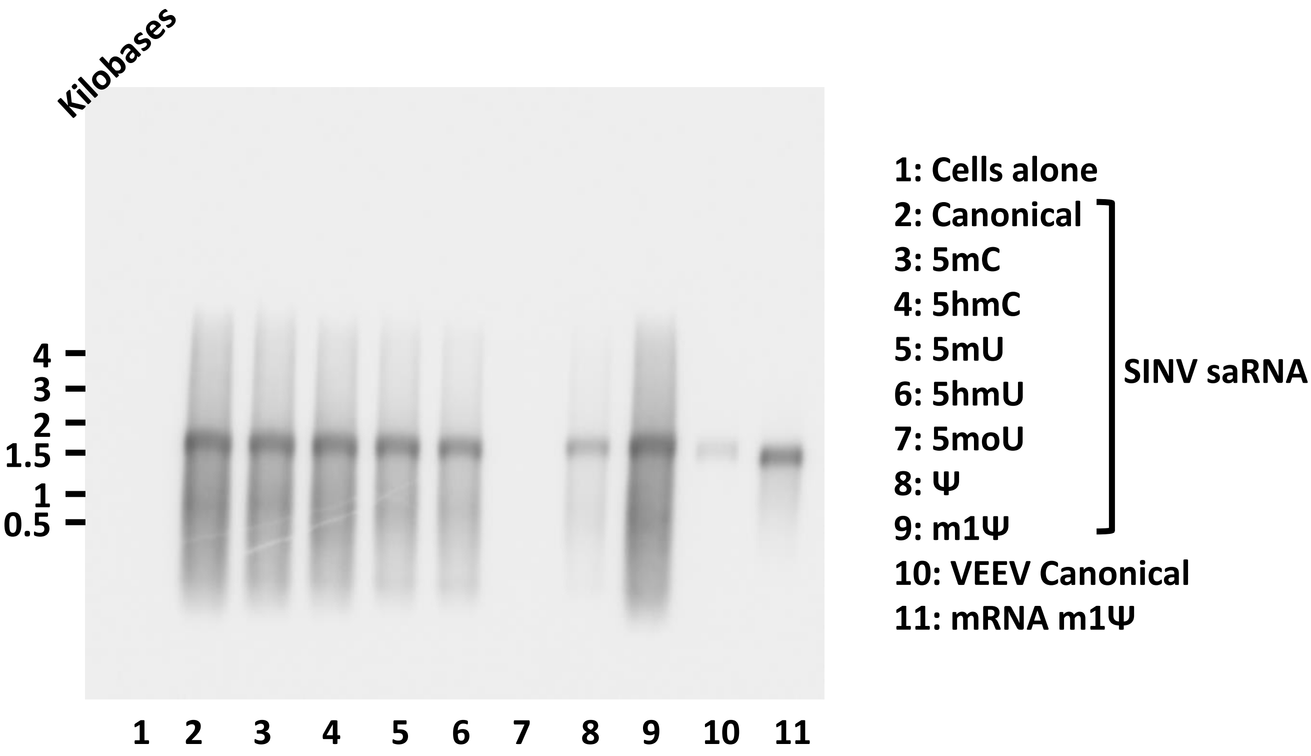

Figure S6
OVA-specific RNA levels in HEK293T cells in vitro. OVA RNA/LNP formulations described in Figure 4 were transfected into HEK293T cells, and RNA isolated for analysis by Northern blot analysis using a probe specific to OVA open reading frame.
